# Effects of resistance training on gait velocity and knee adduction moment in knee osteoarthritis patients: a systematic review and meta-analysis

**DOI:** 10.1038/s41598-021-95426-4

**Published:** 2021-08-09

**Authors:** Shuoqi Li, Wei Hui Ng, Sumayeh Abujaber, Shazlin Shaharudin

**Affiliations:** 1 Exercise and Sports Science Programme, School of Health Sciences, Universiti Sains Malaysia, 16150 Kota Bharu, Kelantan Malaysia; 2 Klinik Kesihatan Putrajaya Presint 9, Kementerian Kesihatan Malaysia, 62300 Putrajaya, Wilayah Persekutuan Putrajaya Malaysia; 3 School of Rehabilitation Sciences, The University of Jordan, Amman, Jordan

**Keywords:** Musculoskeletal system, Risk factors, Therapeutics

## Abstract

The systematic review aimed to analyze the effects of resistance training in knee osteoarthritis (OA) rehabilitation from a biomechanical perspective. A meta-analysis was performed to determine the potential benefits of resistance training on patients with knee OA. Relevant studies based on the inclusion and exclusion criteria were selected from CENTRAL, PubMed, Scopus, and Web of Science databases inception to August 2020. Outcome measures included gait velocity and knee adduction moment (KAM). The mean differences of the data with a 95% confidence interval were analyzed using STATA 15.1 software The search identified eight studies that satisfied all the inclusion criteria, in which 164 patients were involved in gait velocity studies and another 122 patients were part of KAM studies. Analysis of the pooled data showed that resistance training significantly improved the gait velocity in patients with knee OA (*p* < 0.01, z = 2.73), ES (95% CI) = 0.03 (0.01, 0.06) m/s. However, resistance training had no significant effect on improving KAM in patients with knee OA (*p* = 0.98, z = 0.03), ES (95% CI) = 0.00 (− 0.16, 0.16) percentage of body weight × height (%BW × Ht). Therefore, resistance training may enhance gait velocity but not KAM in knee OA patients. The protocol was registered at PROSPERO (registration number: CRD42020204897).

## Introduction

Knee osteoarthritis (OA), a progressive degenerative joint disease, is a highly prevalent musculoskeletal condition that affects approximately 10% of men and 13% of women among adults above 60 years old^1^. Knee OA is also one of the most frequent causes of disability in the elderly as it causes functional limitations, persistent pain, muscle weakness, and poor quality of life^2–4^. Patients with knee OA often suffer from difficulties in walking, thus affecting their daily life activities^5^. To improve patients’ quality of life, it is pivotal to strengthen the function of their lower limbs. For this purpose, resistance training has been suggested to increase muscle strength and improve limb function^6^.

With the advancement of modern medical technology, the three-dimensional (3-D) motion gait analysis system can be used to accurately measure the patient’s lower limb dynamic biomechanics. It also confers various advantages such as easy operation, non-invasiveness, and the generation of detailed data. Furthermore, it is a reliable tool to be used in orthopedic biomechanics research, especially for the objective evaluation of treatment effects^7^. An example of its application is the evaluation of the knee adduction moment (KAM) that affects the load distribution of the knee joint^8^. High KAM has been identified as an important predictor of the presence, severity, and progression of knee OA^9,10^. Furthermore, a retrospective study reported significantly higher KAM among patients with knee OA compared to the normal population^11^. Although exercise has been proven to be an effective way to improve KAM in patients with knee OA^12^, the effect of resistance training on KAM remains unclear.

Gait velocity is another commonly used biomechanical indicator that reflects the motor function of the patient’s lower limbs^13^. Several studies have pointed out that resistance training may be able to improve the gait velocity of knee OA patients by increasing the strength of the quadriceps^14–16^. On the contrary, a few others showed that resistance training failed to improve the gait velocity of knee OA patients^17,18^. The difference in outcomes could be possibly due to the small sample size or different interventions in the studies^17,18^. Therefore, to date, the overall effect of resistance training on gait velocity is still unclear.

In the past, published systematic reviews focused on the effects of resistance training on pain levels, stiffness, and physical functions in knee OA patients^19,20^. However, there is a lack of attention on gait velocity and KAM even though they are important indicators to evaluate the intervention effects on this population. Specifically, there is limited empirical evidence in the literature on the effects of resistance training on the lower limb biomechanics of patients with knee OA. Therefore, this systematic review aimed to evaluate the effects of resistance training in patients with knee OA from the perspective of biomechanics, i.e., gait velocity and KAM. The findings of this study would provide a theoretical basis for the application of resistance training in knee OA rehabilitation.

## Materials and methods

### Protocol and registration

#### Data Sources and study selection

A systematic search was conducted in CENTRAL, PubMed, Web of Science, and Scopus databases. Relevant studies up to August 2020 were selected. The search terms used were "Biomechanics", "Gait", "Osteoarthritis", "Arthritis", "KOA", "OA", "Exercise", "Train", and "Training". The detailed search strategy was listed in Appendix A.

Two investigators (S.L., & W.H.N.) independently screened all the titles and abstracts from the databases. Information extracted from each study included the first author, year of publication, study design, age group and gender of patients, adverse events, type of training program, intervention duration, as well as the results of main indicators captured at baseline and final point (Table 1). In the event of any unclear information, the corresponding author of the respective papers was contacted through e-mail to seek further details. Furthermore, whenever a dispute arose between the two investigators, the opinion of a third investigator (S.S.) was sought. A discussion was held to resolve the issue together to reach a consensus. The protocol was registered at PROSPERO (registration number: CRD42020204897).

**Table 1:** Characteristics of included studies.

Study	Design	Mean age (year) ± SD	Sample size	Participants	Eligibility criteria	Adverse events	Exercise intervention	Duration	Results
Foroughi (2011a)	RCT	64.0 ± 7.0	18	Female	Clinical symptoms; Radiographic findings of knee OA	NA	Progressive RT: lower limb musculature, 80% RM, 8 × 3 sets, 3 times/week	6 months	KAM was not significantly different (*p* = 0.355) from baseline for progressive RT group Gait velocity increased significantly (*p* = 0.001) in progressive RT group over time with a percentage change of 8.6%
Foroughi (2011b)	RCT	66.0 ± 8.0	26	Female	Radiographic findings of knee OA	knee and back pain (n = 1)	Progressive RT: lower limb musculature, 80% RM, 8 × 3 sets, 3 times/week	6 months	KAM was not significantly different (*p* = 0.437) from baseline for progressive RT group
Brenneman (2018)	Cohort	60.8 ± 6.3	40	Female	Clinical symptoms	NA	Isometric RT: lower limb musculature,1 hour, 3 times/week	12 weeks	Gait velocity was not significantly different (*p* = 0.163) from baseline
Sled (2010)	Cohort	63.0 ± 9.7	40	Male (58%) Female (42%)	Clinical symptoms; Radiographic findings of knee OA	NA	Theraband RT: hip musculature, 3–4 times/week	8 weeks	KAM did not change significantly over time (*p* > 0.05)
Henriksen (2017)	RCT	64.9 ± 9.1	24	Male (8%) Female (92%)	Clinical symptoms; Radiographic findings of knee OA	NA	Facility-based RT: trunk, hip and knee musculature, 1 hour, 3 times/week	12 weeks	Both KAM and gait velocity were not significantly different (*p* > 0.05) from baseline for exercise group
DeVita (2018)	RCT	58.1 ± 6.5	15	Male (33%) Female (67%)	Clinical symptoms; Radiographic findings of knee OA	NA	Facility-based RT: quadriceps, 60–85% 3RM, 10 × 3 sets, 3times/week	12 weeks	Gait velocity was significantly increased (*p* = 0.014) by 3% in the training group compared to a 3% decrease in the control group
King (2008)	Cohort	48.4 ± 6.5	14	Male (86%) Female (14%)	Radiographic findings of knee OA	NA	Isokinetic RT: knee extensors and flexors, 1: 60°/s, 90°/s and 120°/s, 60% peak torques, 10 × 3 sets 2: 180°/s, maximum effort, 15 × 3 sets, 3 times/week	12 weeks	Both KAM and gait velocity did not change significantly over time (*p* > 0.05)
Davis (2019)	RCT	62.3 ± 7.1	53	Male (53%) Female (47%)	Radiographic findings of knee OA	NA	Progressive RT: 20 min, knee extension, knee flexion, and hip abduction exercises, 2.5 times/week	4 weeks	Gait velocity increased (*p* = 0.001) in combined group from baseline to 4-weeks with a percentage change of 3.6%

*RT* resistance training; *KAM* knee adduction moment; *OA* Osteoarthritis; RCT randomized controlled trials; *NA* not available; *RM* repetition maximum.

### Inclusion and exclusion criteria

The inclusion and exclusion criteria for the systematic review were set. Firstly, interventional studies involving resistance training among patients with knee OA were included. The intervention, however, must occur for at least four weeks. Furthermore, all relevant studies that met these two criteria must also report at least one of the variables being investigated, either gait velocity and/or KAM. In addition, the gait biomechanics must be tested using three-dimensional (3-D) motion analysis. All randomized controlled trials (RCT) and non-RCT studies published in English were included, whereas abstracts, conference proceedings, or presentations were excluded.

### Quality assessment

The PEDro scale^21^ was used to evaluate the quality of all the included RCT. The PEDro scale has a total of 11 questions. A score of six or above indicates high quality RCT^22^. The quality of non-RCT studies was evaluated with the "Quality Assessment Tool for Before-After (Pre-Post) Studies with No Control Group" (NIH) scale^23^. NIH has 12 questions in total. Based on the total scores, the quality of each study was classified as “poor”, “fair” or “good”.

### Risk of bias assessment

The sensitivity analysis was performed by excluding one by one study to determine the stability of the results of the meta-analysis. The publication bias of the study was analyzed by using a funnel plot. In addition, Egger's test and Begg's test were used to assess the publication bias.

### Data analysis

Analysis of the data was conducted using STATA 15.1 for Windows (version 21 Nov 2017, STATA, College Station, TX, USA). The descriptive statistics for gait velocity and KAM were presented as mean ± standard error. The mean difference before and after intervention with 95% confidence intervals (95%CIs) was calculated as a measure of treatment effect. The calculation was performed based on the method outlined in the Cochrane Handbook (https://training.cochrane.org/handbook).

Next, the Q test and I^2^ statistics were used to estimate the statistical heterogeneity between the studies. The type of model used depended on the Q test results. A random-effect model would be used if there was considerable heterogeneity as shown by *p* < 0.05 or I^2^ > 50%. Otherwise, a fixed-effect model would be used. The methodology in this systematic review was based on the guidelines in the Meta-Analysis Report Quality statement^24^.

## Results

### Eligibility of studies

**Figure 1:**
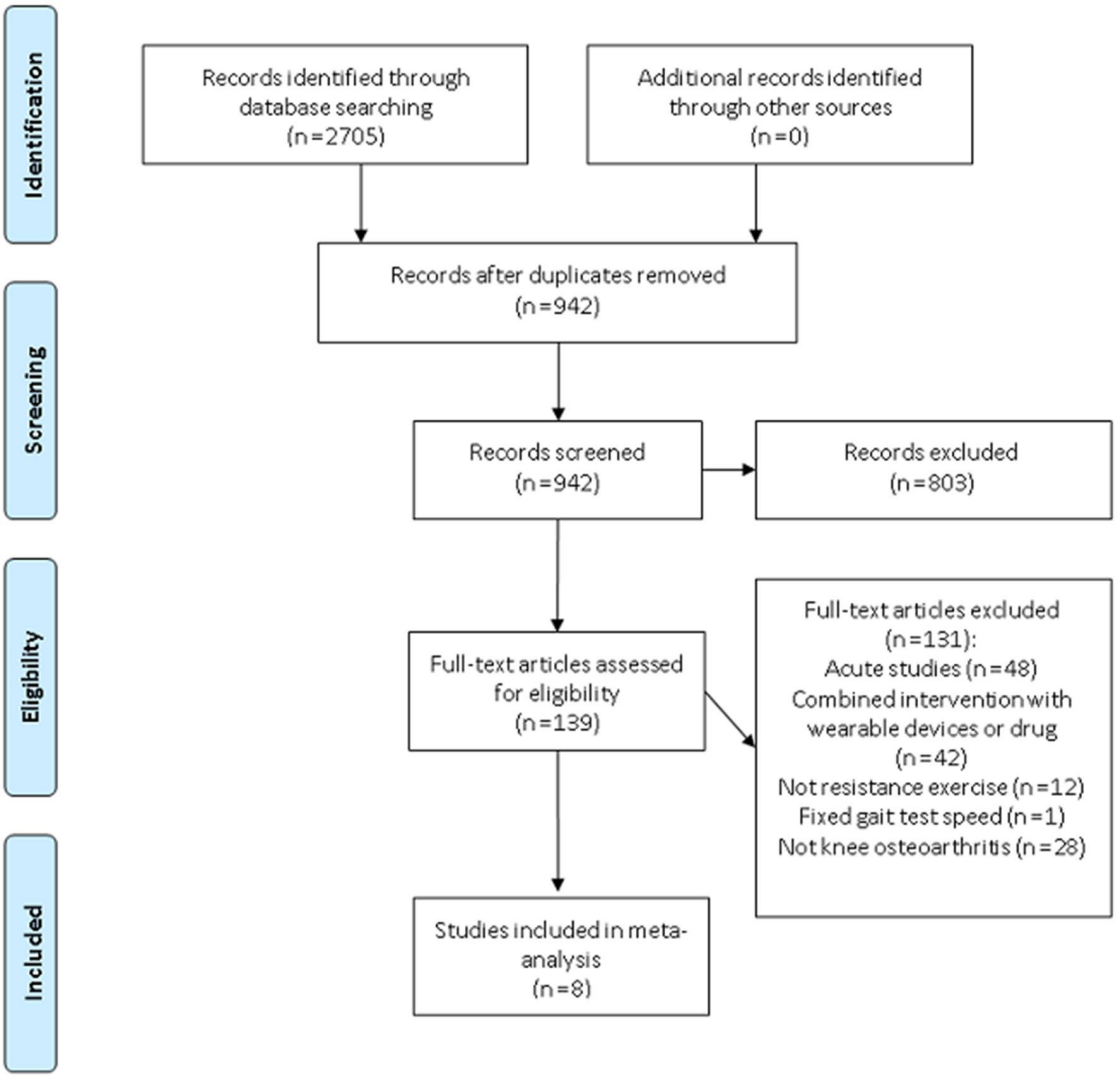
Flow diagram showing the process of identifying and selecting the relevant studies.

The Cohen kappa coefficient between the search by the two investigators was 0.888. A total of eight studies that evaluated the effect of resistance training on the lower limb biomechanics of knee OA patients were analyzed, of which five were RCT and the remaining three were non-RCT (Fig. 1). All of the studies met the pre-determined inclusion criteria. All the included studies reported the baseline and final data after the intervention. However, all the RCTs only had one resistance training group. Due to the small sample size of RCT and the inconsistent protocol of the control group, this review was presented as a single-arm meta-analysis (i.e., without a control group). All the selected studies had obtained prior ethical approval from respective institutions. Among the studies, five studies assessed KAM, while six studies evaluated gait velocity (Table 1). A total of 230 patients (70 men and 160 women) were included, with an average age of 60.9 years. The shortest intervention period of resistance training was four weeks, whereas the longest was six months. All included studies performed 3-D motion gait analysis to measure KAM and gait velocity. The extracted data from the studies are shown in Appendix B.

### Quality assessment

For all the included RCT studies^14–17,25^, the total scores of the PEDro scale were higher than 5 points, thus defined as high quality. Based on the NIH scale, the overall quality rating of the included non-RCT studies^18,26,27^ was "Good" (Table 2).

**Table 2:** Depiction of the risk of bias assessment for included studies.

PEDro Scale		1	2	3	4	5	6	7	8	9	10	11	Total
Foroughi (2011a)		Y*	Y	N	Y	Y	N	N	N	Y	Y	Y	6/10
Foroughi (2011b)		Y*	Y	N	Y	Y	N	N	N	Y	Y	Y	6/10
Henriksen (2017)		Y*	Y	Y	Y	N	Y	Y	Y	Y	Y	Y	9/10
DeVita (2018)		Y*	Y	N	Y	N	Y	N	Y	Y	Y	Y	7/10
Davis (2019)		Y*	Y	N	Y	N	N	Y	Y	Y	Y	Y	7/10

NIH Pre-Post Tool	1	2	3	4	5	6	7	8	9	10	11	12	Total
Brenneman (2018)	Y	Y	Y	NR	Y	Y	Y	NR	N	Y	Y	NA*	8/11
Sled (2010)	Y	Y	Y	N	Y	Y	Y	NR	N	Y	Y	NA*	8/11
King (2008)	Y	Y	Y	NR	Y	Y	Y	NR	Y	Y	Y	NA*	9/11

*Y* yes; *N* no; *NA* not applicable; *NR* not reported. * Not included in total score.

### Quantitative synthesis

**Figure 2﻿:**
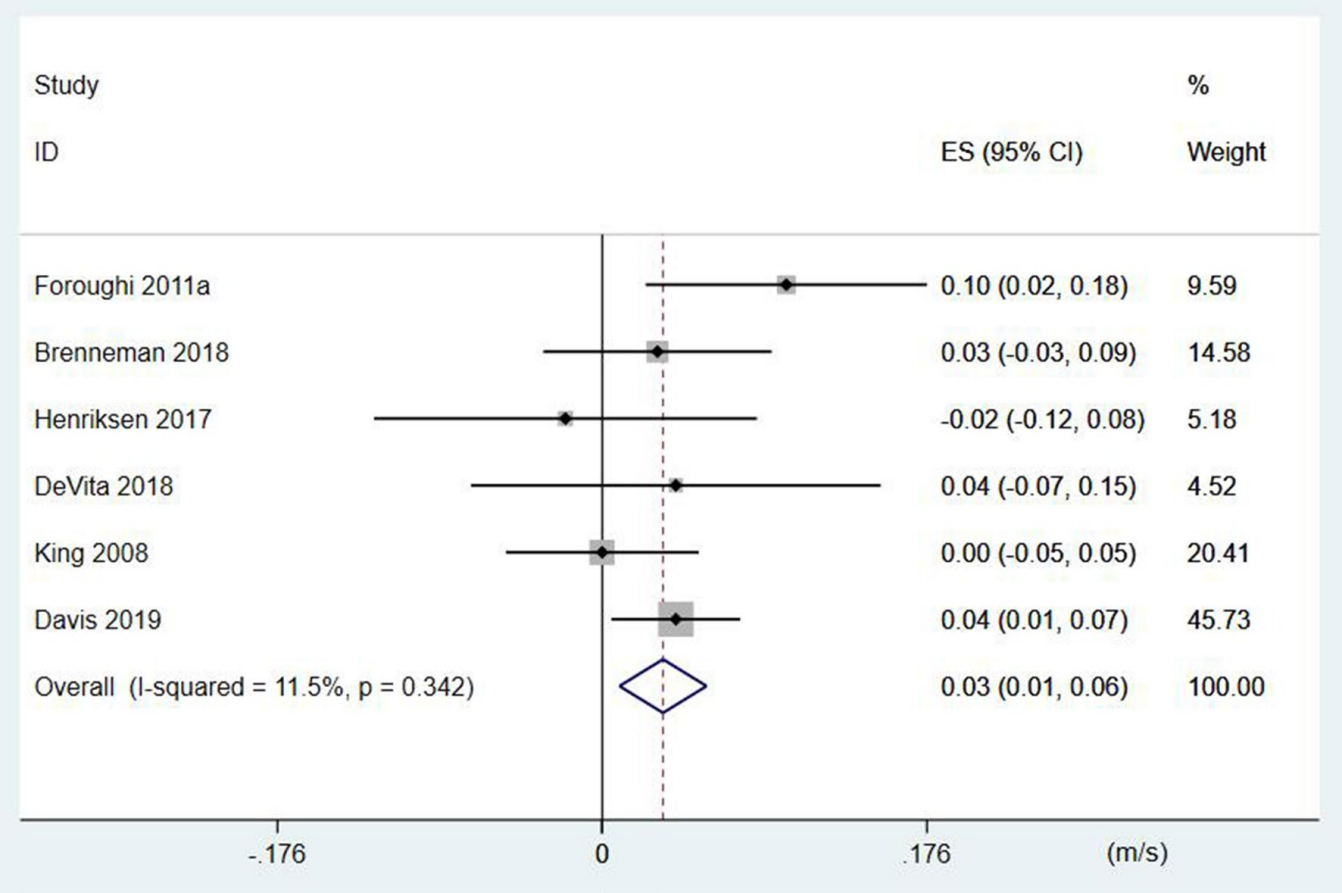
The forest plot of the effect size for studies assessing the effect of resistance exercise on the gait velocity of patients with knee osteoarthritis. The summary effect estimates for the individual studies are indicated by the gray rectangles, with the size of the rectangles proportional to the study weight. The lines represent 95% CI. The overall summary effect estimate and 95% CI are indicated by the diamond shape.

Six studies evaluated the effects of resistance training on gait velocity in patients with knee OA (n = 164). The results of the one-arm meta-analysis indicated that resistance training significantly improved the gait velocity in patients with knee OA (*p* < 0.01, z = 2.73), ES (95%CI) = 0.03 (0.01, 0.06) m/s (Fig. 2). Besides, the heterogeneity test results did not show significant heterogeneity between the studies (*p* = 0.34, I^2^ = 11.5%).

**Figure 3:**
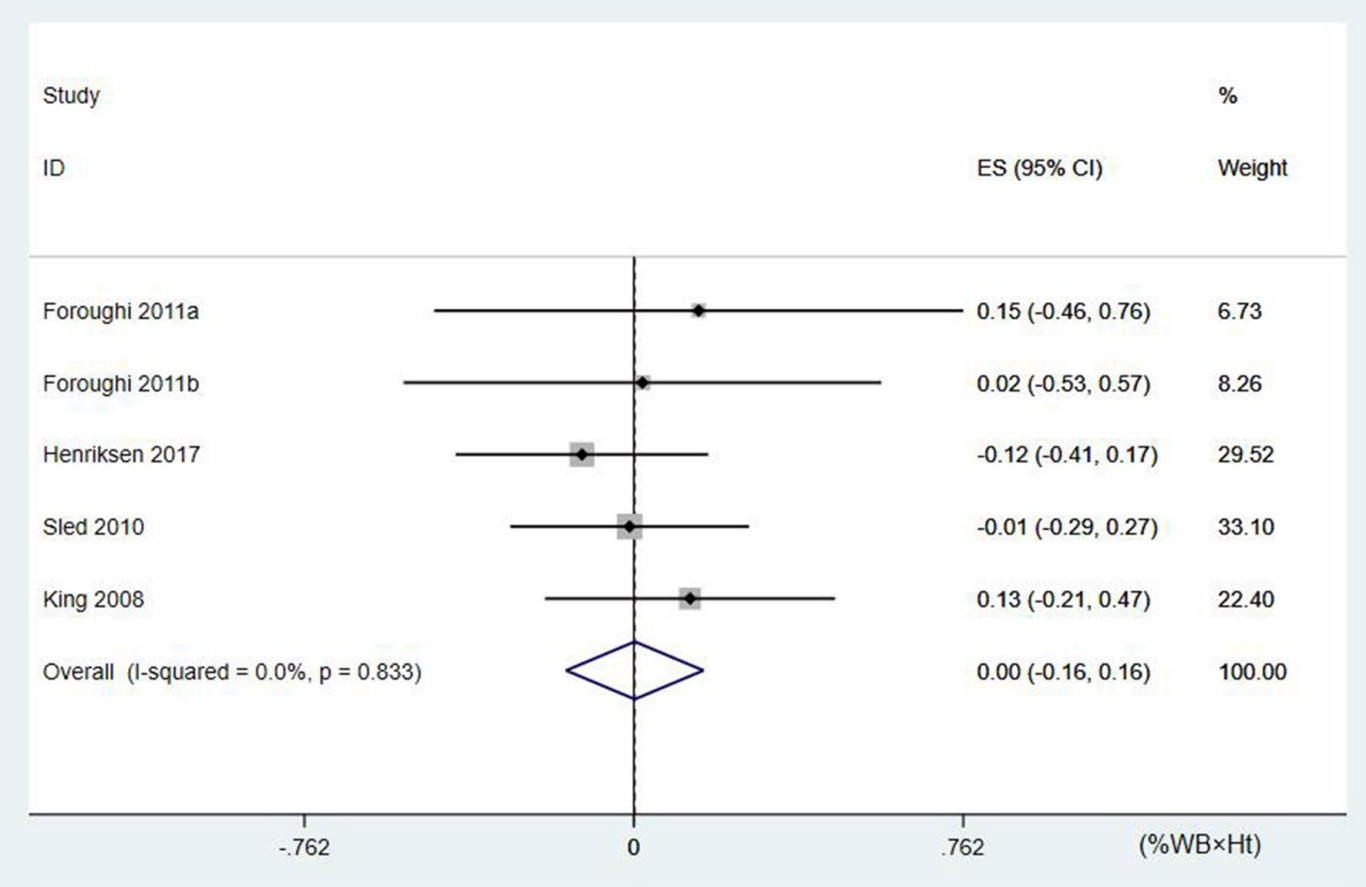
The forest plot of the effect size for studies assessing the effect of resistance exercise on the knee adduction moment (KAM) of patients with knee osteoarthritis. The summary effect estimates for individual studies are indicated by the gray rectangles, with the size of the rectangles proportional to the study weight. The lines represent 95% CI. The overall summary effect estimate and 95% CI are indicated by the diamond shape.

However, using a similar analytical approach, the five studies that evaluated KAM in patients with knee OA (n = 122) did not show the same effectiveness of resistance training (*p* = 0.98, z = 0.03), ES (95% CI) = 0.00 (− 0.16, 0.16) percentage of body weight × height (%BW × Ht) (Fig. 3). Furthermore, there was also no heterogeneity between the studies (*p* = 0.833, I^2^ = 0%).

### Sensitivity analysis

**Figure 4:**
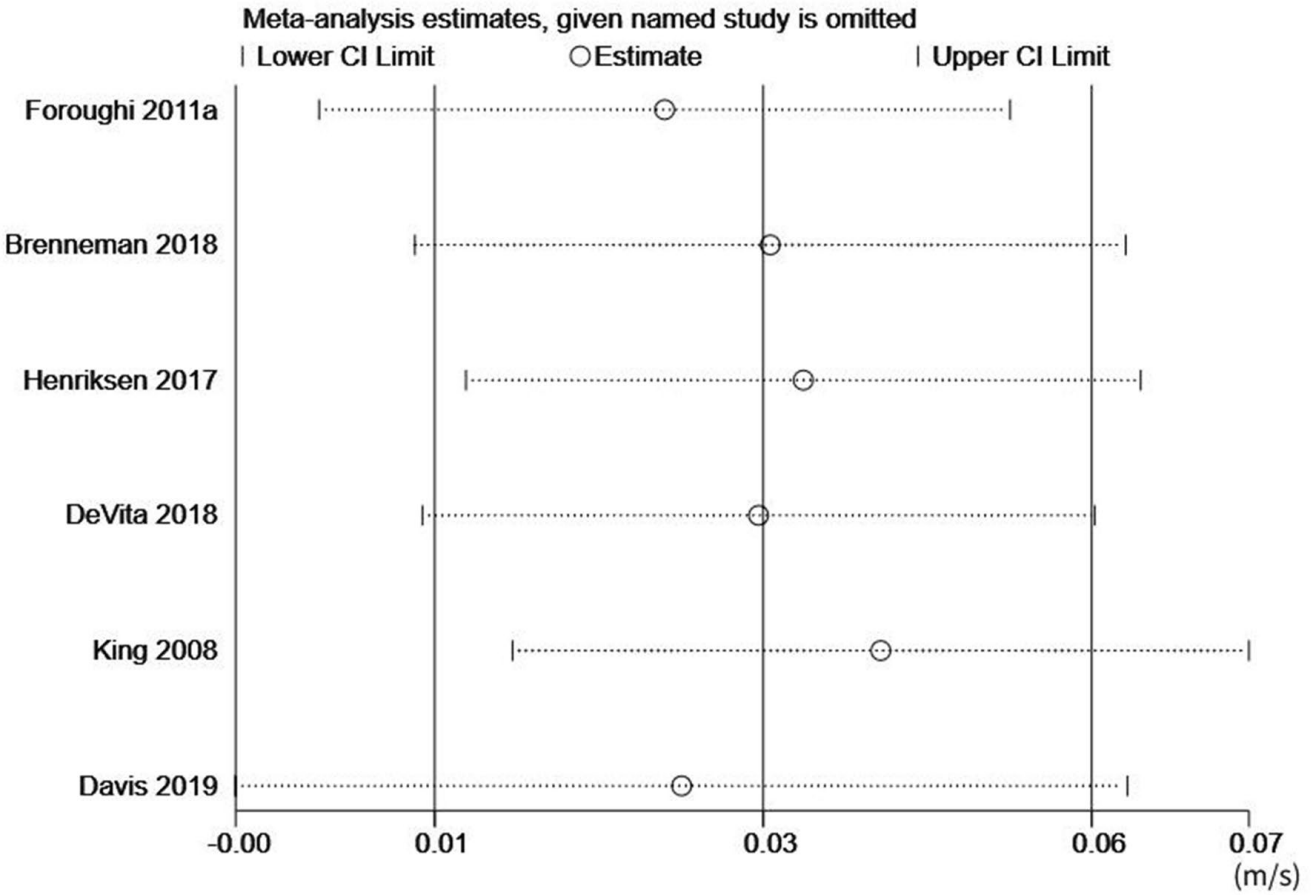
Sensitivity analysis for gait velocity.

**Figure 5:**
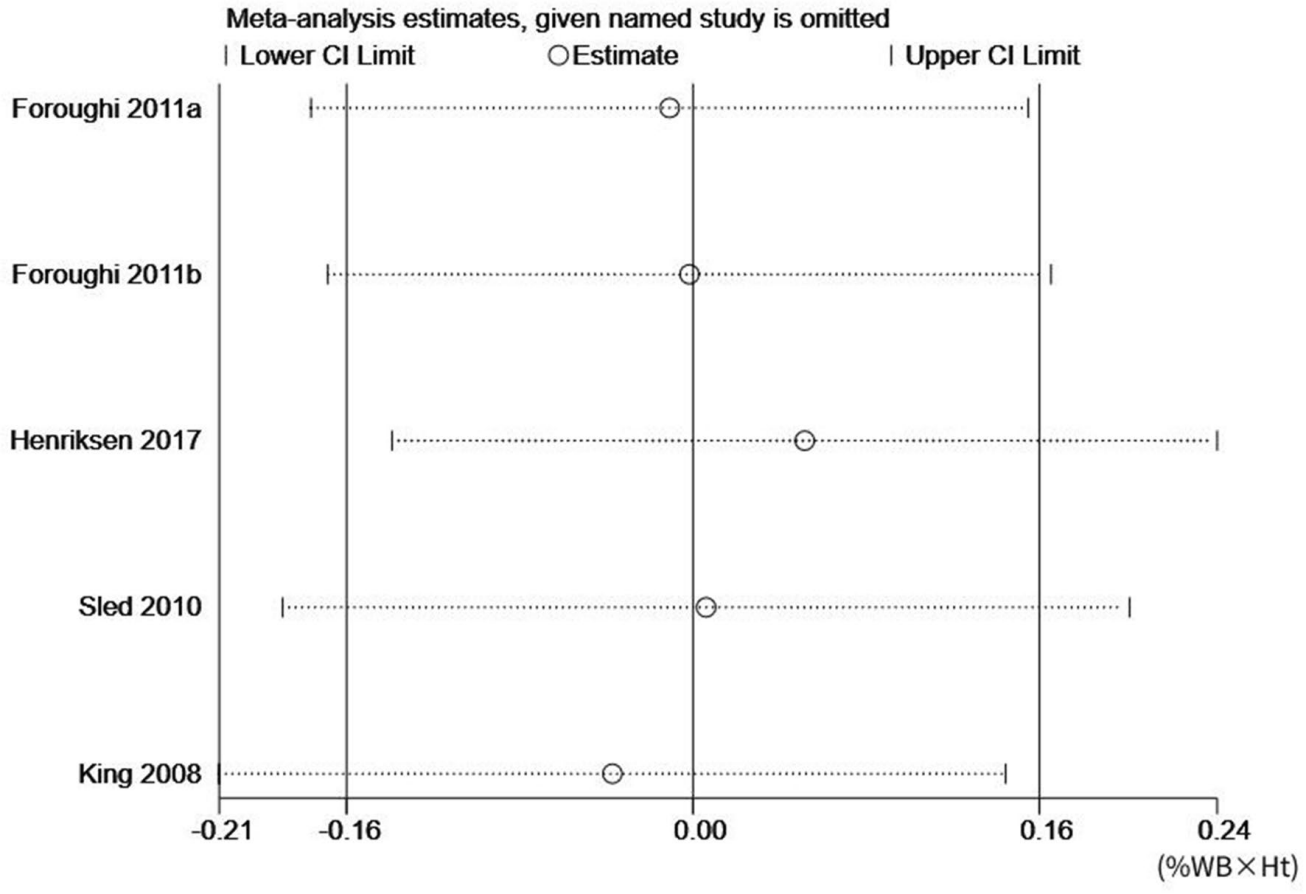
Sensitivity analysis for knee adduction moment (KAM).

Sensitivity analysis was conducted by alteration of the analysis model, selection of effect size, and exclusion of individual articles. Although no heterogeneity was reported among the studies, the sensitivity analysis was still conducted to ensure the accuracy and stability of the data. Based on the sensitivity analysis, each included study had a high degree of agreement with the centerline. Even after deleting any of the studies, the combined effect size did not substantially change the relationship between gait velocity and KAM, thus indicating that the studies have outstanding stability. Figures 4 and 5 outline the sensitivity analysis for gait velocity and KAM, respectively.

### Analysis of publication bias

**Figure 6:**
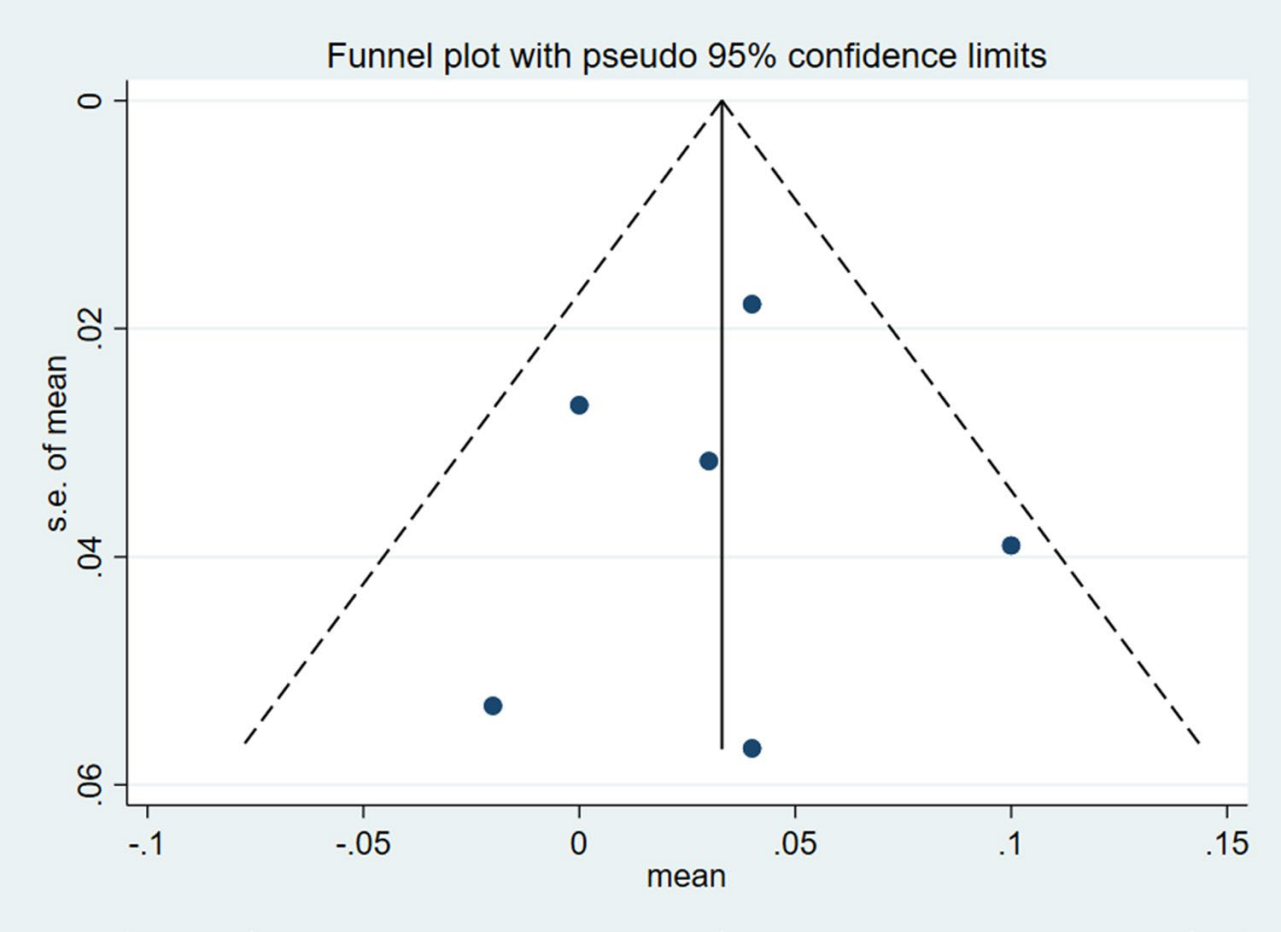
Funnel plot of publication bias for the effect of resistance exercise on the gait velocity.

**Figure 7:**
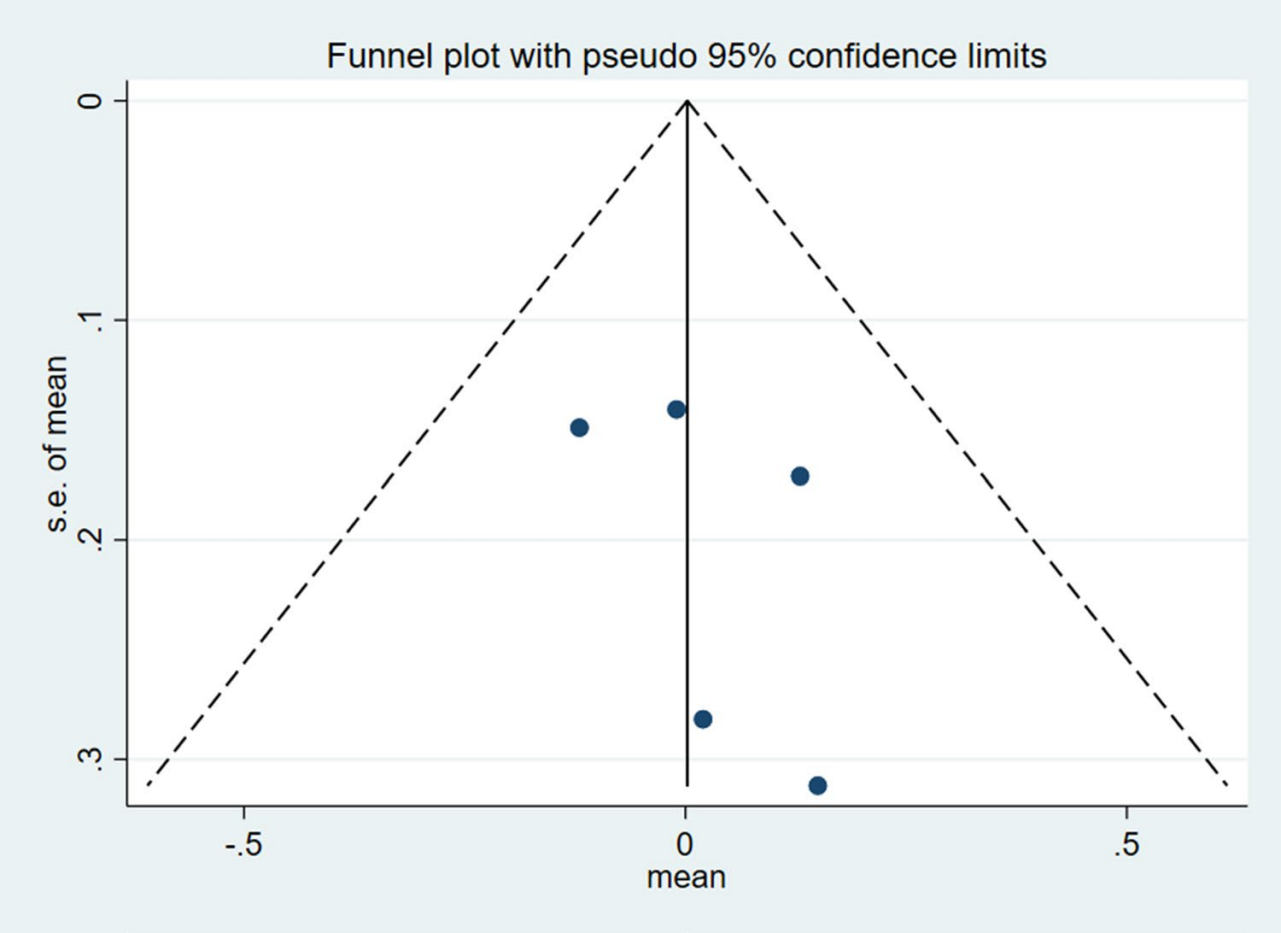
Funnel plot of publication bias for the effect of resistance exercise on the gait knee adduction moment (KAM).

As only eight studies were included in the analysis, the sample size was relatively small to determine the effects of resistance training on patients with knee OA. However, as the total sample size was close to the minimum requirement of an analysis using a funnel plot, the analysis was still considered acceptable to reflect publication bias to a certain extent. A study by Lu et al.^28^, demonstrated the feasibility of funnel analysis for studies with a small sample size. Figure 6 illustrates the funnel chart of the effect of resistance training on the gait velocity of patients with knee OA. Additionally, the results from the Egger's and Begg's tests also indicated no significant publication bias (Egger's test: *p* = 0.934, t =  − 0.09; Begg's test: *p* = 0.851, z = 0.19). Figure 7 displays a funnel chart of the effect of resistance training on KAM among patients with knee OA. Similarly, the results of Egger's and Begg's tests also showed no significant publication bias (Egger's test: *p* = 0.123, t = 2.13; Begg's test: *p* = 0.142, z = 1.47).

## Discussion

This meta-analysis aimed to determine the effects of resistance training on gait velocity and KAM in patients with knee OA. Six articles evaluated the effects of resistance training on gait velocity while five articles assessed the effects of resistance training on KAM. The meta-analysis showed that resistance training significantly improved the gait velocity in knee OA patients. This finding was echoed by another published study in which gait velocity was strongly related to motor function especially among older adults^29^.

Apart from that, a two-center RCT showed that the intervention group that received quadriceps strengthening exercise demonstrated a significant increase in gait velocity by 3% compared to the 3% reduction in the control group, with a large effect size of 0.98^15^. Similarly, Davis et al.^16^ reported a significant increase in gait velocity among knee OA patients after receiving four weeks of progressive resistance (e.g., knee extension, knee flexion, and hip abduction) and balance exercises. It has been established that resistance training could increase quadriceps strength and its neural control, thus further improving walking velocity among the patients^30^. Moreover, numerous studies have also indicated that strength training could reduce pain and disability and produced increased self-walking velocity^8,31,32^. A previous systematic review on walking ability among knee OA patients showed that exercise therapies improved the total distance and gait velocity in general^33^. The therapies encompassed a multitude of exercises including muscle-strengthening, balance, cycling and Tai Chi. Besides, several studies also reported a correlation between pain, muscle strength, gait velocity and the risk of falling^33–37^.

On top of that, this review also revealed that progressive resistance exercise improved the pain level and muscle strength in patients with knee OA^16,27^. This is parallel with the findings of the RCT by Jorge et al.^38^. In other words, gait velocity can be improved after a series of resistance exercise training. However, the relationship between muscle strength and gait velocity warrants further investigation. A previous systematic review has also shown that slow walking decreased the amplitude of gait biomechanics parameters including joint moments and ground reaction force^39^. Moreover, patients with knee OA have been recommended to walk slowly as a possible method to reduce the loads exerted at the knees^40^.

On the contrary, there are also a few studies that reported no significant difference in the gait velocity after a series of resistance exercise training^17,18^. Henrikson et al.^17^ detected no changes in gait velocity and KAM in patients with OA following 12 weeks of individualized neuromuscular exercise program. The program consisted of thrice-weekly strength and coordination exercises prescribed by a trained physiotherapist that involved the trunk, hips, and knees^17^. Nevertheless, the lack of effectiveness might be because these exercises were not specifically aimed at improving walking biomechanics, as discussed in previous studies^14,32^.

Similarly, the absence of improvement in gait velocity was also reported by Brenneman & Maly^18^ in a non-RCT study that utilized a Yoga-based strengthening exercise. The study focused on the effects of a 12-week of strengthening exercise (i.e., lunges, static squat, and gluteal bridge) on hip, knee and ankle biomechanics during gait^18^. Proper posture and alignment were emphasized during the exercises. Even though the average gait velocity increased from 1.14 to 1.17 m/s following the intervention, the changes were not statistically significant. However, the study did not mention the severity of knee OA of each subject and this might play a role in the baseline measurement and the overall results.

The results from our meta-analysis showed that resistance training had no significant effects on KAM in patients with knee OA (n = 122). Theoretically, it was proposed that increased strength of major lower limb muscles might reduce KAM, increase hip adduction moment and slow the progression of OA^25^. In many studies, KAM has been established as a strong predictor of OA progression due to its correlation with the load distribution within the knee joint^25,41^. Moreover, individuals with OA were found to have higher KAM compared to healthy participants^26,42^. Greater KAM was also associated with thinner tibiofemoral cartilage, another indicator of the rate of OA progression^43^.

In a study by Sled et al.^26^, no improvement in KAM was detected following eight weeks of hip abductor strengthening home program in patients with medial compartment OA. This could be attributed to the co-existing biomechanical alteration such as trunk movement^40^. Lateral trunk lean is considered a gait strategy that affects the KAM^40^. It was suggested that increasing muscle strength of the hip abductor could help stabilize the pelvis, thus decreasing the lateral trunk lean towards the stance limb and increasing the moment arm at the knee. As a result, any reduction in KAM from the resistance exercise would have been nullified^26^. On a similar note, an increase in the mediolateral trunk sway was found to reduce the KAM by 65% in a healthy population^40^. However, Foroughi et al.^25^ showed that despite 50% improvement of hip abductors’ strength in patients with knee OA following high-intensity resistance training, no changes in KAM were observed.

Next, Lloyd et al.^45^ suggested that co-contraction of quadriceps and hamstring muscles supports and stabilizes the knee joint, thus subsequently reduces the varus thrust during gait. Varus alignment and varus thrust are both correlated with KAM^46^. Some studies claimed that the strength of the lower limb muscle groups is not the major contributor to KAM, instead other factors such as varus alignment^40,47^, walking velocity^48^, and pain level^47^ contributed more to joint loading. However, among knee OA patients, an increased quadriceps strength following 12-week high-intensity resistance training^27^ or functional and individualized neuromuscular exercise therapy^17^, did not lead to any improvement in the walking KAM.

Furthermore, evidence suggested that the effects of resistance training on KAM might be more prominent in other more demanding tasks than gait^49^. Thorstensson et al.^49^ found that 8 weeks of exercise intervention significantly reduced KAM during the one-leg rise, as compared to no difference of KAM detected during gait. Therefore, they suggested further investigation of KAM using one-leg rise or other functional movements that are more sensitive to show greater deviation in KAM than gait^49^. More importantly, further research is warranted to identify the contributing factors of KAM during functional movement to reduce medial compartment joint loading^26^. For every 20% increment of KAM, the risk of OA progression has been shown to increase six-fold^50^. Similarly, increment of KAM by 8% increased the risk of developing chronic knee pain four years later^51^. Overall, the pooled results from this review showed that resistance training was effective in improving gait velocity and reducing pain, but it was not effective in reducing the abnormal loading on the knee joint, especially KAM during gait.

## Study limitations

Only eight studies met the inclusion criteria of this review, and not all of them were RCTs. This reflects a lack of high-quality literature on the topic. Additionally, there was a considerable variation in the resistance training programs applied in each study, with the duration of interventions ranging from 4 to 24 weeks. Moreover, the participants in the included studies suffered from all different grades of knee OA based on the KL radiographic grades. Hence, the different severity of knee OA might confer a variation effect on the gait biomechanics analysis.

## Recommendations for future studies

Long-term RCTs with larger sample sizes are needed to identify factors associated with disease progression to customize a more effective exercise plan for OA patients^41^. These studies should also compare the various elements of resistance training including the type, dosage, intensity, and volume of exercise as well as the muscle groups involved. Such information is vital to better understand the effect of the training on the KAM during gait, as well as during more demanding functional tasks such as stair-climbing or standing up from a chair. Additionally, the effects of strengthening exercise on pelvic stability, medial and lateral trunk sway in gait biomechanics should be investigated further^26,44^. Moreover, future studies should also explore the incorporation of a specific gait retraining program in the strengthening exercise regime to enhance the effect of KAM in gait biomechanics^25,48^. To address the current limitation, attention should be focused on specific grades of OA to produce more accurate findings. Lastly, further studies should investigate the influence of other potentially moment-modifying factors (e.g., toe-out angle, lateral trunk lean, varus-valgus knee laxity, bracing, insole wedges, and knee malalignment) as well as increased muscle strength^52^ on normalizing joint loading to determine other crucial biomechanical factors.

## Conclusion

This review summarized the effects of resistance training on the gait velocity and KAM of patients with knee OA. Resistance training was found to improve gait velocity, but not the KAM of the OA patients. To identify factors that can reduce the risk of knee OA progression and improve the quality of life of knee OA patients, further studies on the effects of gait-related functional training towards KAM are warranted.

## Supplementary Information

Supplementary Information 1.

Supplementary Information 2.

## Data Availability

The data is available from the corresponding author upon request.
