# Supplementary material for: Effects of resistance training on gait velocity and knee adduction moment in knee osteoarthritis patients: a systematic review and meta-analysis
Source: Sci Rep. 2021 Aug 9;11:16104. doi: 10.1038/s41598-021-95426-4 (PMC8352951; doi:10.1038/s41598-021-95426-4)
Supplement: Supplementary file 1 — Supplementary Information 1. [file 41598_2021_95426_MOESM1_ESM.docx]

**Appendix A**

Search strategy

| **Databases** | **Search strategy** | **Results** | |
| --- | --- | --- | --- |
| Scopus | #1: Title-Abs-Key ("biomechanics" or "gait")  #2: Title-Abs-Key ("Osteoarthritis" or "arthritis" or "KOA” or "OA")  #3: Title-Abs-Key ("exercise" or "train" or "training")  #4: #1 and #2 and #3  Limiters - Published Date: 20080101-20201231 | 293734  430519  1802190  1311 |  |
| Pubmed | #1: [Title/Abstract] "biomechanics" or "gait"  #2: [Title/Abstract] "Osteoarthritis" or "arthritis" or "KOA" or "OA"  #3: [Title/Abstract] "exercise" or "train" or "training"  #4: #1 and #2 and #3  Filters: Publication date from 2008/01/01 to 2020/1/31 | 70837  245790  646089  322 |  |
| Web of Science | #1: TOPIC: ("biomechanics" or "gait")  #2: TOPIC: ("Osteoarthritis" or "arthritis" or "KOA" or "OA")  #3: TOPIC: ("exercise" or "train" or "training")  #4: #1 and #2 and #3  Refined by: PUBLICATION YEARS: (2020-2008)  Indexes=SCI-EXPANDED, SSCI, CCR-EXPANDED,  IC Timespan=All years | 97475  333392  729127  697 |  |
| CENTRAL | #1: ("biomechanics" or "gait"):ti,ab,kw  #2: ("Osteoarthritis" or "arthritis" or "KOA" or "OA"):ti,ab,kw  #3: ("exercise" or "train" or "training"):ti,ab,kw  #4: #1 and #2 and #3  Year: All years | 10483  36456  149651  375 |  |
