# Supplementary material for: Effects of resistance training on gait velocity and knee adduction moment in knee osteoarthritis patients: a systematic review and meta-analysis
Source: Sci Rep. 2021 Aug 9;11:16104. doi: 10.1038/s41598-021-95426-4 (PMC8352951; doi:10.1038/s41598-021-95426-4)
Supplement: Supplementary file 2 — Supplementary Information 2. [file 41598_2021_95426_MOESM2_ESM.docx]

Appendix B

| Gait velocity (m/s) | Baseline | | Final | | Difference | |
| --- | --- | --- | --- | --- | --- | --- |
|  | Mean±SD | n | Mean±SD | n | Mean±SD | n |
| Foroughi (2011a) | 1.10±0.17 | 19 | 1.20±0.17 | 19 | 0.10±0.17 | 19 |
| Brenneman (2018) | 1.14±0.21 | 40 | 1.17±0.19 | 40 | 0.03±0.20 | 40 |
| Henriksen (2017) | 1.37±0.25 | 24 | 1.35±0.27 | 24 | -0.02±0.26 | 24 |
| DeVita (2018) | 1.43±0.21 | 15 | 1.47±0.23 | 15 | 0.04±0.22 | 15 |
| King (2008) | 1.12±0.11 | 14 | 1.12±0.10 | 14 | 0.00±0.10 | 14 |
| Davis (2019) | 1.12±0.10 | 53 | 1.16±0.15 | 53 | 0.04±0.13 | 53 |
| KAM (%BW×Ht) |  |  |  |  |  |  |
| Foroughi (2011a) | 2.54±1.41 | 19 | 2.69±1.30 | 19 | 0.15±1.36 | 19 |
| Foroughi (2011b) | 2.63±1.26 | 20 | 2.65±1.26 | 20 | 0.02±1.26 | 20 |
| Henriksen (2017) | 3.78±0.82 | 24 | 3.66±0.59 | 24 | -0.12±0.73 | 24 |
| Sled (2010) | 2.97±0.87 | 40 | 2.96±0.90 | 40 | -0.01±0.89 | 40 |
| King (2008) | 3.30±0.72 | 14 | 3.43±0.49 | 14 | 0.13±0.64 | 14 |

KAM= Knee adduction moment; %BW×Ht= percentage of body weight×height.
